# Antibacterial Effects of Essential Oils of Seven Medicinal-Aromatic Plants Against the Fish Pathogen *Aeromonas veronii* bv. *sobria*: To Blend or Not to Blend?

**DOI:** 10.3390/molecules26092731

**Published:** 2021-05-06

**Authors:** Manolis Mandalakis, Thekla I. Anastasiou, Natalia Martou, Sofoklis Keisaris, Vasileios Greveniotis, Pantelis Katharios, Diamanto Lazari, Nikos Krigas, Efthimia Antonopoulou

**Affiliations:** 1 Hellenic Centre for Marine Research, Institute of Marine Biology, Biotechnology and Aquaculture, 71500 Heraklion, Greece; mandalakis@hcmr.gr (M.M.); theanast@hcmr.gr (T.I.A.); katharios@hcmr.gr (P.K.); 2 Laboratory of Animal Physiology, Department of Zoology, School of Biology, Faculty of Sciences, Aristotle University of Thessaloniki, 54124 Thessaloniki, Greece; natamart@bio.auth.gr (N.M.); keisaris@bio.auth.gr (S.K.); 3 Institute of Industrial and Forage Crops, Hellenic Agricultural Organization Demeter, 41335 Larisa, Greece; vgreveni@mail.com; 4 Laboratory of Pharmacognosy, School of Pharmacy, Faculty of Health Sciences, Aristotle University of Thessaloniki, 54124 Thessaloniki, Greece; dlazari@pharm.auth.gr; 5 Institute of Plant Breeding and Genetic Resources, Hellenic Agricultural Organization Demeter, P.O. Box 60458, 57001 Thessaloniki, Greece

**Keywords:** essential oil combinations, Greek native MAPs, natural products, antimicrobial activity, synergistic effects, bacterial pathogens, aquaculture

## Abstract

Despite progress achieved, there is limited available information about the antibacterial activity of constituents of essential oils (EOs) from different medicinal-aromatic plants (MAPs) against fish pathogens and the complex interactions of blended EOs thereof. The present study aimed to investigate possible synergistic antimicrobial effects of EOs from seven Greek MAPs with strong potential against *Aeromonas veronii* bv. *sobria,* a fish pathogen associated with aquaculture disease outbreaks. The main objective was to evaluate whether blending of these EOs can lead to increased antimicrobial activity against the specific microorganism. A total of 127 combinations of EOs were prepared and their effect on *A. veronii* bv. *sobria* growth was tested in vitro. We examined both the inhibitory and bactericidal activities of the individual EOs and compared them to those of the blended EOs. The vast majority of the investigated combinations exhibited significant synergistic and additive effects, while antagonistic effects were evident only in a few cases, such as the mixtures containing EOs from rosemary, lemon balm and pennyroyal. The combination of EOs from Greek oregano and wild carrot, as well as the combinations of those two with Spanish oregano or savoury were the most promising ones. Overall, Greek oregano, savoury and Spanish oregano EOs were the most effective ones when applied either in pure form or blended with other EOs.

## 1. Introduction

The bacterium *Aeromonas veronii* Hickman-Brenner et al. 1988 poses a serious threat worldwide to the aquaculture industry as it is responsible for mortality outbreaks in a variety of farmed fish, including Nile tilapia (*Oreochromis niloticus* Linnaeus 1758) [1], African catfish (*Clarias gariepinus* Burchell 1822), rajputi (*Puntius gonionotus* Bleeker 1850), rui (*Labeo rohita* Hamilton 1822), catla (*Catla catla* Hamilton 1822), and shole (*Channa striatus* Bloch 1793) [2], Chinese longsnout catfish (*Leiocassis longirostris* Günther 1864) [3], loach (*Misgurnus anguillicaudatus* Cantor 1842) [4] and European seabass (*Dicentrachus labrax* Linnaeus 1758) [5]. The symptoms of the affected fishes typically include exophthalmia, ulcers and haemorrhagic septicaemia. In particular, *A. veronii* bv. *sobria* has caused many problems for the culture of European seabass (*D. labrax*) in Greece, especially during the last decade [5]. Antibiotics, probiotics, prebiotics, algae, phages, minerals, nanoparticles, plants and essential oils have all been recruited to deal with severe infections caused by *Aeromonas* spp. To date, the tested vaccines and the alternative treatments used have only partially addressed this problem [6,7,8].

The increasing resistance of fish pathogens to conventional antibiotics and the societal demand for less harmful fish products and concomitant processes from environmental/sustainability viewpoint has channeled research towards alternative treatments of bacterial infections. In particular, there is growing interest about the antibacterial properties of natural resources and natural products, such as essential oils (EOs) of medicinal-aromatic plants (MAPs) [9].

Due to their anti-bacterial, antifungal, antiviral, and insecticidal properties combined with strong antioxidant activity, EOs stand out as promising alternative natural agents in animal husbandry and fish farming for the prevention or even the treatment of several infectious diseases [10,11,12,13,14,15,16]. EOs from various MAPs are utilized as powerful antimicrobial and antioxidant additives for fishery products’ preservation increasing their shelf-life [10]. Additionally, EOs have also been incorporated into fish feeds with promising results to mitigate bacterial infections during rearing [11,12,13]. EOs often act as inhibitors of toxic bacterial metabolites, they can hinder bacterial growth [9,14], or serve as powerful natural antioxidants competing with synthetic ones [15]. EOs are generally considered as protective agents preventing or mitigating the oxidative damage from reactive oxygen species (ROS) in animals; the latter, is typically triggered by stressful conditions and generally results in altered fish immune functions [9,13,14,15,16].

Despite the research progress in testing antibacterial efficacy of EOs from various MAPs and of their isolated major components, there are limited studies focusing on potential antagonistic, additive or synergistic effect of mixed EOs from different MAPs or other sources [17,18,19,20]. Therefore, it remains largely unclear whether the antimicrobial effects of individual EOs is retained or even increase once these are blended with EOs from other MAPs [19,20].

To date, there are only few studies investigating the effect of EOs and their individual components against *A.* *veronii* bv. *sobria* [21]. Additionally, there is only limited knowledge regarding the complex interactions of blended EOs from different MAPs against the specific pathogen [21]. In this context, the primary aim of the present study was to investigate possible synergistic antimicrobial effects of mixed EOs from selected MAPs of Greece against *A. veronii* bv. *sobria*. Our investigation was specifically focused on EOs from seven MAPs that showed the greatest inhibitory activity against bacterial fish pathogens in a previous screening study [21]. To this end, a total of 127 combinations of EOs from the seven selected MAPs were prepared and were tested in vitro against *A. veronii* bv. *sobria*. A secondary aim of this study was to evaluate whether blending of these EOs can result in an enhancement of antimicrobial activity against the selected bacterial fish pathogen. To address this aim, we systematically examined the inhibitory and bactericidal activities of EOs blends and compared them to those of pure counterparts.

## 2. Results

### 2.1. Chemical Composition of the Tested Essential Oils

The GC-MS analysis of pure EOs from the seven MAPs indicated 49 main constituents in total. The relative concentrations of individual components are presented in Table S1 (Supplementary Materials). Among the Lamiaceae members, savoury, Greek oregano and Spanish oregano had several compounds in common, with *p*-cymene (6.5 to 11.9%) and γ-terpinene (5.3 to 34.0%) being the main constituents after carvacrol (32.8% to 72.0%). Rosemary was found to be rich in eucalyptol (45.0%), camphor (11.5%) and α-pinene (8.8%), while pennyroyal contained pulegone (47.6%) and piperitenone (33.0%) as main components. In the wild carrot, isoeugenol methyl ether (14.8%), α-pinene (20.5%) and β-himachalene (21.6%) were detected at high levels. Lemon balm demonstrated a distinct chemical composition among EOs with citronellal (10.2%), γ-muurolene (12.6%) and β-caryophyllene (27.7%) being the main components.

The GC-MS analysis of seven bi- and tripartite EO blends with the highest inhibitory activity indicated 26 main constituents in total. The concentrations of individual components of these blended preparations of EOs are presented in Table 1.

Carvacrol (33.15–73.77%), γ-terpinene (4.20–10.53%) and *p*-cymene (7.10–9.71%) were the major components found in all the highly effective bi- and tripartite blends of EOs against *A. veronii* bv. *sobria*. Blend #11, which consisted of Greek oregano and Spanish oregano (1:1), showed the highest cumulative amount of carvacrol (73.77%), while the percentage content of this compound in individual EOs was lower (i.e., 42.0% and 72.0% in EOs of Spanish oregano and Greek oregano, respectively. Carvacrol was detected in higher amounts in blends #8, #16 and #31 (56.36%, 56.78% and 65.78%, respectively) compared to its amounts found in the individual EOs of savoury (32.8%) and Spanish oregano (42.0%). 

It is interesting that in the blend #35 consisting of EOs from Greek oregano, rosemary and Spanish oregano (1:1:1), carvacrol was in less amount (33.15%) than those of the individual essential oils of Greek oregano (72.0%) and Spanish oregano (42.0%). Moreover, this blended preparation was rich in eucalyptol (20.15%), a major compound of pure rosemary essential oil (45.0%). In blends #10 and #38 (containing wild carrot EO), α-Pinene was one of their major compounds; however, this was in less amounts (18.44% and 13.14%, respectively) compared to that of the individual EO oil of wild carrot (20.5%).

### 2.2. Antibacterial and Bactericidal Activity of Blended Essential Oils

**Figure 1:**
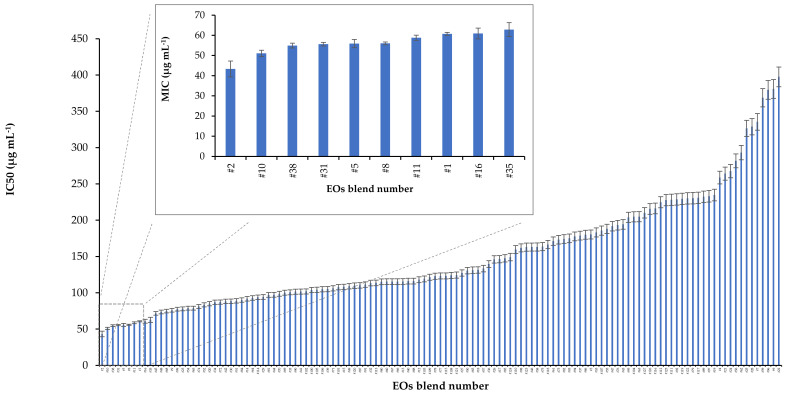
Inhibitory concentrations (IC_50_ in μg mL^−1^) of 127 blends of EOs from seven Greek aromatic-medicinal plants against the fish pathogen *Aeromonas veronii* bv. *sobria.* The inset highlights the IC_50_ values of the top-10, most effective blends. The composition of the seven EOs in each blend is shown in Table S2 (Supplementary Materials).

**Figure 2:**
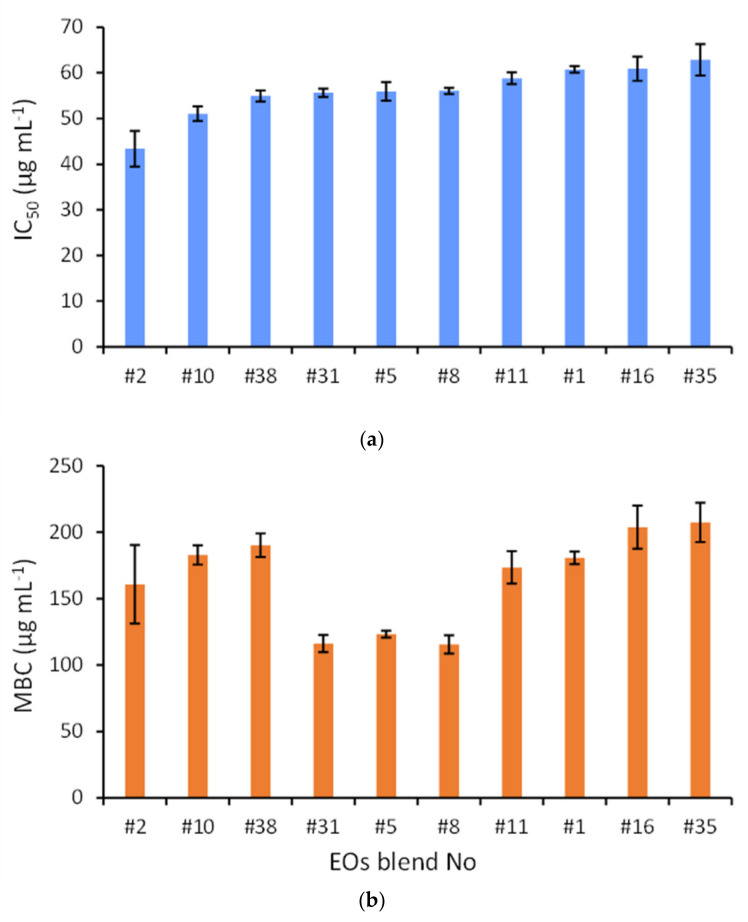
(**a**) Inhibitory Concentration (IC_50_ in μg mL^−1^; upper panel) and (**b**) Minimum Bactericidal Concentration (MBC in μg mL^−1^; lower panel) for the top-10 most effective blends of essential oils against the fish pathogen *Aeromonas veronii* bv. *sobria*. The exact composition of the seven essential oils in each blend is shown in Table S2.

The seven EOs were effectively mixed at various proportions and a total of 127 blends were prepared and tested against *A. veronii*. The growth inhibitory concentration (IC_50_) of the different blends varied by an order of magnitude (43.4 to 397.5 μg mL^−1^), providing a median value of 123.3 μg mL^−1^ (Figure 1). With regard to the top-10, most effective blends, the IC_50_ diverged much less and varied from 43.4 to 62.8 μg mL^−1^ (Figure 2a). Despite this limited variability, statistically significant differences were evident in the inhibitory activity of specific blends. In particular, the solution #2, which corresponded to pure EO from savoury (Table S2, Supplementary Materials) exhibited the highest antibacterial activity (43.4 ± 3.9 μg mL^−1^) with a statistically significant difference in comparison with all other blends tested (t-test, *p* < 0.05). The blend #10 containing EOs from Greek oregano and wild carrot in equal proportions provided the second strongest inhibition against *A. veronii*, with its IC_50_ value (51.1 ± 1.6 μg mL^−1^) also being statistically different from those of the other mixtures (Table 2). The rest of the top-10 blends (i.e., #38, #31, #5, #8, #11, #1, #16, #35) presented similar antibacterial activities, with respective IC_50_ values having a relative standard variation of about 5% and the majority of pairwise differences barely reaching statistical significance (Table 3).

Regarding the composition of the top-10, most effective blends, it is worth stressing that seven of them contained EO from Greek oregano, while another six included EO from Spanish oregano, but none of them contained pennyroyal or lemon balm. Indeed, the EOs of Spanish oregano (solution #5; IC_50_: 55.9 ± 2.0 μg mL^−1^) and Greek oregano (solution #1; IC 60.7 ± 0.7 μg mL^−1^) were among the single-note EOs featured in the list of most effective EOs. With respect to bipartite composites of EOs (Table 1), the most prominent one was a combination of Greek oregano with wild carrot (blend #10), followed by another three blends which also contained Greek oregano or Spanish oregano (blend #8, #11, #16). Only three tripartite composites were featured in the top-10 list (Table 1), with all of them containing Greek oregano and Spanish oregano in combination with savoury (blend #31) or rosemary (blend #35) or wild carrot (blend #38).

More complicated blends containing four to seven different EOs showed comparatively decreased effectiveness against *A. veronii* and they were totally absent from the top-10 list. The ten blends with the highest inhibitory activity were further tested for their ability to cause complete eradication of *A. veronii*. The minimum bactericidal concentrations (MBC) of the examined blends ranged from 116 to 207 μg mL^−1^ (Figure 2b) and they were two to four times higher than the corresponding IC_50_ values (Figure 2a). Blends #5, #31 and #8 exhibited marginal differences in terms of bactericidal activity and their MBC values (116 to 123 μg mL^−1^) were significantly lower compared to all other blends. Interestingly, the top-2 hits (#31 and #8) were blends of Greek oregano, savoury and Spanish oregano, while the third hit (#5) was the only consisting of a pure EO (i.e., Spanish oregano). Among the other seven blends, the MBC values varied by less than 9% (161 to 207 μg mL^−1^) and they were at least 30% higher compared to the group of the three most effective combinations.

In Figure 2a, the ten best-performing blends for bacterial growth inhibition are ranked hierarchically from the smallest to the highest IC_50_. When contrasted to Figure 2b, it is apparent that a similar trend of gradually increasing bactericidal activity (MBC) applies for the blends #2, #10, #38, #16 and #35. On the other hand, the blends #31, #5, #8 as well as the blends #11 and #1 representing different combinations of Greek oregano, Spanish oregano and savoury deviated from the IC_50_ pattern and showed comparatively lower MBC values than anticipated.

**Figure 3:**
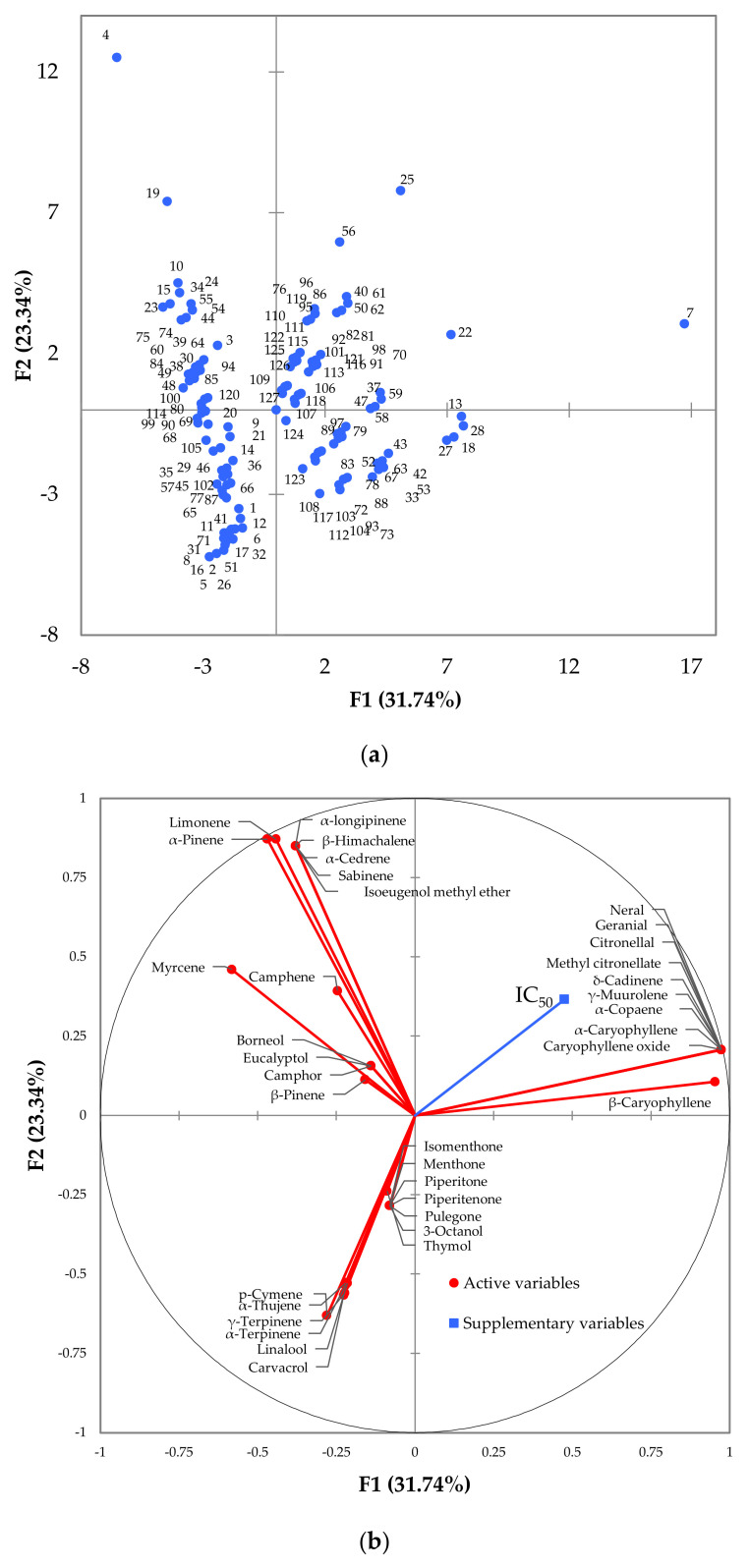
Principal component analysis scores plot (**a**) and loadings plot (**b**) derived from the compositional data of the 127 essential oils’ blends examined (see Table S1 Supplementary Materials). The blue dots reflect the differences in the chemical composition of individual blends, while the red vectors represent the 36 chemical compounds of the essential oils that were used as active variables. The half maximal inhibitory concentration (IC_50_) of the blends against *Aeromonas veronii* bv. *sobria* is also projected on the loading plot (blue vector; supplementary variable).

The differences in the chemical composition of pure EOs and their blends as well as the interrelations of inhibitory activity with chemical constituents were further evaluated by principal component analysis (PCA). The first two principal components (PC1 and PC2) explained 31.7% and 23.3% of the total variance present in the dataset (Figure 3). Interestingly, eight out of the top-10, most effective blends were clustered together in the outer part of the lower left quadrant of PCA scores plot (Figure 3a). The same area of PCA loadings plot was populated by carvacrol, γ- and α-terpinene, *p*-cymene, α-thujene, γ-terpinene and linalool, all of them being major shared constituents of Greek oregano, savoury and Spanish oregano (Table S1, Supplementary Materials). More importantly, the IC_50_ vector was positioned in the diagonally opposite quadrant, implying the strong influence of those compounds to the increase of inhibitory activity against *A. veronii* bv. *sobria* (i.e., lower IC_50_ values for higher levels of the specific compounds in the blends). The rest two blends (#10, #38) remained in the left side of the scores plot (i.e., opposite to IC_50_ vector), but at the upper quadrant. Both of them contained wild carrot, the main compounds of which (e.g., α-pinene, β-himachalene, isoeugenol methyl ether, limonene) were also placed at the upper left panel of the loadings plot.

### 2.3. Assessment of Synergistic Antimicrobial Action of Essential Oils in Blends

**Figure 4:**
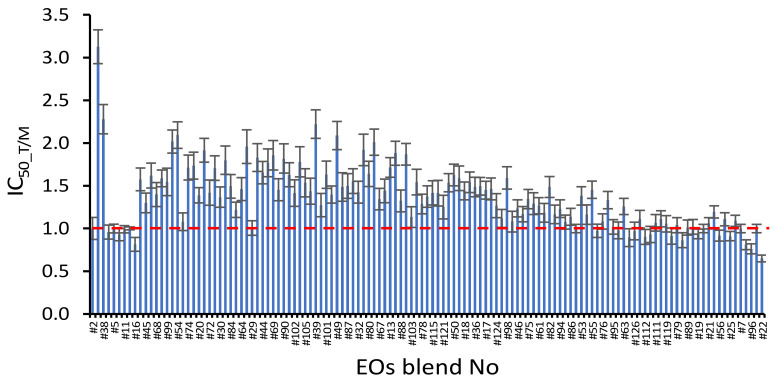
Ratios between theoretical and measured 50% inhibitory concentration (IC_50_T/M_) of the investigated 127 essential oils (EOs) blends. The datapoints lying on the dashed line (IC_50_T/M_ = 1) reflect an additive antibacterial activity of EOs against *Aeromonas veronii* bv. *sobria*, while the datapoints above and below the dashed line represent synergistic and antagonistic interactions of EOs, respectively.

By taking into account the proportions and IC_50_ values of the seven EOs, the theoretical IC_50_ of blends were calculated (on the basis of additive effects) and compared to the experimentally measured values. More specifically, the ratio between theoretical and measured inhibitory concentration (IC_50_T/M_) was calculated for all 127 blends and the results were used to evaluate the additive (IC_50_T/M_ = 1), synergistic (IC_50_T/M_ > 1) or antagonistic effects (IC_50_T/M_ < 1) in the different EOs combinations (Figure 4). For 46 blends the IC_50_T/M_ values (0.81 to 1.29) were statistically indistinguishable from 1, implying additive antibacterial interactions between mixed EOs.

More interestingly, a total of 71 combinations presented IC_50_T/M_ ratios significantly higher than 1 (1.19 to 3.13), indicative of synergistic antibacterial effect, while antagonistic interactions between EOs were inferred only for three blends (IC_50_T/M_: 0.65 to 0.81). By far the highest value was observed for the 1:1 mixture of Greek oregano and wild carrot (blend #10; IC_50_T/M_ = 3.1), for which the measured IC_50_ was 68% lower than the theoretically calculated IC_50_. Similarly, the second highest ratio was found for the 1:1:1 mixture of Greek oregano, wild carrot and Spanish oregano (blend #38; IC_50_T/M_ = 2.3), which presented 56% difference between measured and theoretical IC_50_. Both blends were included in the top-10 most potent blends against *A. veronii* bv. *sobria*.

## 3. Discussion

Several pure EOs from single MAP species have been tested to date for their antimicrobial activity against microbial pathogens of aquaculture environments with promising results [9,21,22,23]. However, there is limited knowledge regarding the antimicrobial activity of blended EOs [19,24,25]. The particular interest for blending relies on the concept that the combination of EOs from two or more MAPs may result in a product with greater bacteriostatic or bactericidal efficacy than each individual EO alone. This is possible due to the synergistic effects that may arise from the coupling of chemical components from the different EOs. On the other hand, the combination of EOs may also lead to simply additive or even antagonistic effects. In this context, the activities of various EO mixtures are typically examined and the results are usually contrasted with the effects of single EOs and/or dominant compounds thereof [19,24,25]. In general, any detected interaction (synergistic, additive or antagonistic) between two compounds of a single EO or between two or more EOs in a mixture depends on the concentrations of individual components or single EOs [26,27] and the overall susceptibility of the bacteria tested [26]. Although several investigated blends of EOs have shown antimicrobial potency against animal pathogens with promising results [28,29,30,31,32], the studies dealing with blends of EOs tested against microbial pathogens of the aquaculture sector still remain scarce to date [19,24,25,33].

### 3.1. Inhibitory Activity: Single-Note Essential Oils and Their Combinations

Among the seven pure EOs extracted from MAPs and tested herein, those being phenolic/carvacrol-rich were the most effective against *A. veronii* bv. *sobria*. More specifically, the EOs from savoury, Spanish oregano and Greek oregano were the most effective growth inhibitors of this particular fish pathogen. In addition, their antibacterial activities were greater than those found for the majority of the 127 combinations of EOs tested and they were ranked in the top-10 most potent agents overall. The enhanced potency of those three pure EOs was most likely due to their greater abundance in highly effective compounds, such as carvacrol (32.8% to 72.0%), γ-terpinene (5.3 to 34.0%) and *p*-cymene (6.5 to 11.9%) [21].Among the bi- or tripartite mixtures, the combination of Greek oregano with wild carrot in 1:1 ratio (blend #10) showed the best antibacterial activity, exhibiting stronger inhibitory effects compared to the single EOs, as also indicated by the ratio of theoretical IC_50_ to the measured one. More specifically, the presence of wild carrot in this carvacrol-rich blend seems to play a synergistic role in antibacterial potency, implying a possible positive interaction of its individual constituents such as α-pinene, isoeugenol methyl ether and β-himachalene (Table S1 and Table 1), with those of Greek oregano. This was also the case for blend #38, where the combination of Greek oregano with Spanish oregano and wild carrot (1:1:1) resulted in a highly effective mix, which showed higher inhibitory activity than the pure individual EOs (i.e., synergistic effects). The antibacterial properties of *Daucus carota* EO against both Gram positive and negative bacteria, as well as fungi, have already been investigated [21,34,35], with some studies [34,36] reporting isoeugenol methyl ether as one of the most active compounds. Moreover, it has been reported that the simultaneous presence of high levels of both hydrophobic and hydrophilic compounds, such as hydrocarbons and phenols, can more readily affect the integrity and permeability of the cell membrane of microorganisms [37]. Further studies on the antimicrobial properties of the individual EO components in pure form and in combination are warranted to elucidate the efficacy observed in the above-mentioned EOs mixtures.

Unexpectedly, the mixture of Greek oregano, savoury and Spanish oregano (1:1:1, blend #31, which were the most carvacrol-rich EOs was not the most effective blend against the tested bacterial strain. The inhibitory activity of this blend was ranked fourth among all 127 combinations and it was lower than the activity of savoury EO, but higher than those of pure EOs from Greek and Spanish oregano. Interestingly, the 1:1 combination of Greek oregano with Spanish oregano (blend #11) exhibited nearly additive effect (ratio = 0.99), showing slightly higher antimicrobial activity compared to single-note EO of Greek oregano. Similarly, the blend of Greek oregano with savoury (blend #8) and the one composed of Spanish oregano with savoury (blend #16) were deemed to exhibit additive interactions since their IC_50_T/M_ ratios (i.e., 0.93 and 0.81, respectively) were not statistically different from 1 (at *p* < 0.05).

Last but not least, the blend of Greek oregano and Spanish oregano with eucalyptol-rich rosemary (blend #35, 1:1:1) showed the lowest antibacterial activity of the top-10 effective inhibitors, yet exhibiting synergistic effects. Like wild carrot, rosemary is another example of EO that is less effective when used alone, and its major components, like eucalyptol, camphor and α-pinene, seem to interact positively with constituents of other EOs in a blend leading to an increase of the overall antimicrobial activity. Nonetheless, all the aforementioned mixtures were included in the top-10 most efficient blends and they altogether deserve a detailed in vivo investigation in farmed fish to validate their actual efficacy against *Aeromonas veronii* bv. *sobria*.

### 3.2. Bactericidal Properties

The bactericidal activity of the top-10 most effective growth inhibitors (pure EOs and in combination) of *A. veronii* bv. *sobria* was also investigated. Intriguingly, a different pattern compared to the inhibitory activity (IC_50_) was recorded, with blends of Greek oregano with savoury (#8), Greek oregano with savoury and Spanish oregano (#31), and pure Spanish oregano EO (#5) being the most effective. However, this is not surprising considering that carvacrol, a compound highly abundant in these three solutions, is already reported to have lethal effects on several Gram-negative and Gram-positive bacteria, such as *Listeria monocytogenes* (Murray et al. 1926) Pirie 1940, *Campylobacter jejuni* (Jones et al. 1931) Veron & Chatelain 1973, *Escherichia coli* (Migula 1895) Castellani & Chalmers 1919, *Salmonella enterica* (ex Kauffmann & Edwards 1952) Le Minor & Popoff 1987 [32], and *Bacillus cereus* [38,39]. Nevertheless, it is worth noticing that the third most fatal EO was pure Spanish oregano with 42.0% carvacrol content and not Greek oregano containing 72.0% carvacrol. This finding suggests that bactericidal activity of EO blends cannot be predicted solely on the basis of carvacrol content. Hence, other minor constituents and their interactions with the major ones, may play an important role in the germicidal behaviour of these EOs. The beneficial antimicrobial activity of members of the Lamiaceae family, and especially of carvacrol-rich MAPs is widely recognized [9]. Nevertheless, the potential toxic effects of EOs and/or of their chemical components on organisms are dose-dependent [9]. In this context, it is quite likely that the positive effect of EOs administration on fish applied as anti-bacterial agents against pathogens can be achieved at much lower concentrations than their respective toxicity thresholds. The mechanism of antibacterial action of the EOs’ composition either used alone or when mixed in blends deserves to be studied in more detail in order to elucidate the complexity of concomitant interactions [36]. It is worth deciphering why combinations of EOs with a strong individual antimicrobial efficacy, such as Greek oregano or Spanish oregano and savoury, do not actually show synergistic or additive effects when blended, and why -on the other hand- combinations of two or more EOs with individually moderate activity, such as those of wild carrot, rosemary, etc. studied herein, may result in considerably enhanced effects when are combined in blended EOs preparations.

## 4. Material and Methods

### 4.1. Plant Material and Extraction of Essential Oils

The plant material of seven medicinal-aromatic plants originated from the companies Dioscurides (Anarrachi, Ptolemaida-Kozani, Greece), Icaronix (Ikaria Island, Greece) and Vessel Essential Oils (Neo Rysio, Thessaloniki, Greece). The essential oils (EOs) were industrially extracted from the air-dried aerial parts of six perennials herbs of the Lamiaceae family, namely *Melissa officinalis* L., *Mentha pulegium* L., *Origanum vulgare* L. subsp. *hirtum* (Link) A. Terrac., *Rosmarinus officinalis* L., *Satureja thymbra* L., *Thymbra capitata* (L.) Cav., and one of the Apiaceae family (*Daucus carota* L.). The plants were cultivated in northern Greece (Thessaloniki, Ptolemaida, Grevena) and Ikaria Island in south-eastern Greece, except for wild carrot which was harvested from wild habitats (Table 4).

### 4.2. Gas Chromatography-Mass Spectrometry Analysis

The single essential oils for each of the seven investigated MAPs were analyzed on a GCMS-QP2010 (Shimadzu, Kyoto, Japan) gas chromatography-mass spectrometry system to determine their chemical composition. The separation of the compounds was achieved using a HP-5 MS capillary column (30 m × 0.25 mm i.d., film thickness 0.25 μm; Agilent Technologies, Santa Clara, CA, USA). The EOs of the most effective bi- and tripartite blends were analyzed on a GCMS-QP2010 (Shimadzu) gas chromatography-mass spectrometry system to determine their chemical composition. The separation of the compounds was achieved using an INNOWAX fused silica column (30 m × 0.25 mm i.d., film thickness: 0.25 μm; Agilent Technologies).

All the samples were diluted with hexane (1:10, *v/v*). For all samples, the injection volume was 1μL, in split mode (1:10) and the injector temperature was kept at 230 °C; Helium was the carrier gas at a flow rate of 1.0 mL min^−1^. The column temperature for the single-note EOs was programmed at a rate of 4 °C min^−1^ from 50 °C to 290 °C, and at a rate of 3 °C min^−1^ from 50 °C (20 min) to 250 °C for bi- and tripartite blends of EOs. For all samples, the temperatures of the GC–MS transfer line and ion source were maintained at 300 °C and 230 °C, respectively, while ionization was performed in the EI mode (70 eV), and full-scan mass spectra were acquired from m/z 100 to 600. Arithmetic indices for all compounds of single-note EOs were determined using *n*-alkanes as standards [40]. The components of individual/pure EOs and blended EOs were identified on the basis of their mass spectra and their retention indices compared with those listed in NIST21 and NIST107 mass spectral databases [40,41], assisted also by data reported in scientific literature [42]. For all samples, the identity of several components in single or blended EOs was further confirmed by co-chromatography with authentic compounds. The relative percentage amounts of the separated compounds were calculated from the total ion chromatogram by a computerized integrator.

### 4.3. Evaluation of Antibacterial and Bactericidal Activity

The strain *Aeromonas veronii* bv. *sobria* was used in this study to evaluate the antibacterial and bactericidal activity of EOs. The strain was isolated from European seabass (*Dicentrarchus labrax*) farmed in Argolikos Bay, Eastern Peloponnese [5].

Blending was restricted to EOs from seven MAPs that were previously found to possess the most potent inhibitory activity against fish bacterial pathogens [21]. These included Greek oregano, Spanish oregano, savoury, rosemary, wild carrot, pennyroyal and lemon balm. In order to investigate EOs blending in a systematic way and reveal how the proportions of various EOs affect antibacterial activity, a full simplex-centroid experimental mixture design was applied (Statgraphics Centurion 18, Statgraphics Technologies Inc., The Plains, VI, USA). This resulted in 127 combinations with each component varying from 0 to 100% (Table S2, Supplementary Materials).

The ability of each mixture to inhibit the growth of the pathogen *A. veronii*, was evaluated using the broth microdilution method described in our previous studies [21,43]. This was based on the microplate procedure originally presented by Wiegand et al. [44] and it followed CLSI guidelines [45] with some necessary modifications (e.g., Mueller-Hinton broth replaced by Brain Heart Infusion (BHI) medium). In brief, an overnight broth culture of *A. veronii* was diluted with fresh BHI broth of 2× concentration to obtain a turbidity equal to 0.5 McFarland (i.e., ~10^8^ CFU mL^−1^). The bacterial suspension was further diluted 1:100 with 2× BHI to achieve a cell density of ~10^6^ CFU mL^−1^. Each EO was first dissolved in DMSO and then diluted with water to a final stock concentration of 1600 μg mL^−1^ (final DMSO concentration of 10%). Mixing of EOs in varying proportions (Table S2, Supplementary Materials), preparation of eleven two-fold serial dilutions from each blend in 384-well microplates (50 μL in each well; final concentration of 0.39 to 800 μg mL^−1^) and addition of bacterial suspension in BHI broth (50 μL in each well; 1× BHI final concentration) were performed using an automated liquid handling system (Biomek 2000; Beckman Coulter, Fullerton, CA, USA).

Microplates were incubated at 25 °C (optimum growth temperature for *A. veronii*) for 22 h and bacterial growth in each microculture was monitored by measuring optical density at 600 nm (OD600) every 20 min using a microplate reader (Infinite F200 PRO, Tecan GmbH, Grödig Austria). The area under the growth curve (i.e., OD600 vs. time) was integrated for each microdilution assay and the data were used for estimating half maximal concentration of each blended essential oil inhibiting 50% of bacterial growth (IC_50_). All experiments were performed in triplicate and the average IC_50_ of each blend was derived. Growth controls (cell culture without EOs) and sterility controls (BHI broth without cells) were also included in every microplate that was assayed. In addition, treatment of bacterial cells with florfenicol and oxytetracycline, two standard antibiotics that are commonly administered to farmed fishes, were used as positive controls.

Minimum Bactericidal Concentrations (MBC) were determined for the top-10 most effective EO blends showing the lowest IC_50_ values. After repeating serial dilutions and IC_50_ experiments for the specific blends, 10 μL were obtained from the different microcultures and transferred into a 384-well microplate containing fresh BHI medium. The microplate was incubated at 25 °C and bacterial growth was monitored for 22 h, as described above. MBC was determined as the lowest EO blend concentration yielding no bacterial growth. Six replicate measurements of MBC were performed for each blend.

The chemical composition of the 127 blends was calculated using the mixing ratios and the corresponding compositions of pure EOs reported in our recent study [21]. To reveal putative relationships between EOs ingredients and their inhibitory activity against the target bacterial pathogen, a principal component analysis (PCA) was performed. A total of 36 compounds presenting a concentration higher than 0.5% in at least 30% of the 127 blends were used as active PCA variables, while the MIC values were also projected onto the PCA plot as a supplementary variable (i.e., not considered for the computation of the components). The PCA was conducted based on Pearson’s correlation matrix by using the XLSTAT software (version 2016; Addinsoft Inc., New York, NY, USA).

## 5. Conclusions

In this study, individual EOs of seven Greek native MAPs and numerous mixtures thereof in all possible combinations (blends of two to seven MAPs in equal proportions) were assessed for their antimicrobial properties against the fish bacterial pathogen *A. veronii* bv. *sobria*. Additive and synergistic growth-inhibiting properties were observed in most of the mixtures examined, with the EOs blend of Greek oregano and wild carrot exhibiting the greatest synergistic action. On the other hand, antagonistic interactions were evident only in a few mixtures characterized by the combined presence of rosemary, lemon balm and pennyroyal. Greek oregano, savoury and Spanish oregano EOs were the most effective ones when applied either in pure form or blended with other EOs. With regard to their blends, the combination of EOs from Greek oregano and wild carrot, as well as the combinations of those two with Spanish oregano or savoury (all in equal proportions) were among the most potent ones against bacterial growth. Overall, the choice of the best EOs mixture against *A. veronii* bv. *sobria* depends on whether the main purpose is to inhibit its growth or achieve its complete eradication. For growth inhibition, the pure EO from savoury (blend #2) and the mixture of Greek oregano and wild carrot EOs (blend #10) represent the best options. However, blended EOs of Greek oregano and savoury EOs (blend #8), as well as Greek oregano, Spanish oregano and savoury EOs blend (#31) are more efficient in achieving the complete eradication of the study pathogen. If both inhibition and/or complete suppression of bacterial activity is the aim, the blends #5, #8 and #31 are the best ones, showing consistently low values for both IC_50_ and MBC, with Greek/Spanish oregano being the common EO in all three blends, and carvacrol being their most abundant chemical compound. For growth inhibition of the studied pathogen, pure savoury EO and the mixture of Greek oregano and wild carrot EOs represent the best options; for its complete eradication, the blended Greek oregano and savoury EOs as well as Greek oregano, Spanish oregano and savoury EOs blend are more efficient. If both inhibition and eradication are aimed, the two latter blends and pure Spanish oregano EO are highly effective (all with abundant carvacrol content). Undoubtedly, further in vivo tests are required to verify the applicability and effectiveness of this alternative antibacterial agent under real fish farming conditions. In practice, fish feeds supplemented with various levels of the aforementioned best-performing EO blends should be prepared and used in infected fish to reveal whether these natural plant products are effective in farmed fish without side effects for large-scale curative and/or preventive treatments.

## Figures and Tables

**Table 1:** Chemical composition (relative percentage % of the main compounds) of the most effective bi- and tripartite blends of essential oils (EOs) against *Aeromonas veronii* bv. *sobria* after evaluation of 127 preparations using all combinations of EOs of seven Greek native medicinal-aromatic plants. Blend #8: Greek oregano and savoury (1:1); Blend #10: Greek oregano and wild carrot (1:1); Blend #11: Greek oregano and Spanish oregano (1:1); Blend #16: Savoury and Spanish oregano (1:1); Blend #31: Greek oregano, savoury and Spanish oregano (1:1:1); Blend #35: Greek oregano, rosemary and Spanish oregano (1:1:1); Blend #38: Greek oregano, wild carrot and Spanish oregano (1:1:1). For the GC-MS chromatograms of the most effective blended essential oil preparations, see Supplementary Materials Figure S1.

		Bipartite Blends				Tripartite Blends		
No.	Compound ^a^	#8	#10	#11	#16	#31	#35	#38
1	α-Pinene	5.59	18.44		7.44		8.40	13.14
2	α-Thujene	2.23	0.67		3.91	2.82	2.50	1.48
3	Camphene	0.33	0.89	0.21	0.32	0.28	2.34	0.74
4	β-Pinene	0.32	0.97	0.13	0.29	0.24	4.21	0.75
8	β-Myrcene	1.33	2.91	1.34	1.24	1.27	1.58	2.67
9	α-Terpinene	1.34	0.86	1.16	1.25	1.22	1.03	1.22
10	D-Limonene	0.26	3.28		0.21	0.20	1.24	2.32
12	Eucalyptol						20.15	
13	γ-Terpinene	10.53	4.20	5.39	9.25	8.05	4.59	5.46
14	*p*-Cymene	9.71	8.07	8.53	7.10	8.30	7.74	8.78
15	α-Longipinene		1.80					1.27
16	Camphor						3.18	
17	Linalool	0.92	0.28	0.54	1.29	0.99		0.59
18	β-Caryophyllene	4.34	0.91	2.87	5.60	4.54	2.22	2.10
19	1-Terpinen-4-ol	1.00	0.90	0.96	0.85	1.78	1.00	1.16
20	Thymol methyl ether	1.17	0.32	0.35	0.85		0.09	
21	β-Himachalene		1.11					0.73
22	Borneol	0.70	0.50	0.99	0.98	1.02	2.21	0.73
23	Palustrol		5.83					3.83
24	Thymol	1.05	0.02	1.50	0.44	1.01	0.66	0.04
25	Isoeugenol methyl ether		4.26					2.80
26	Carvacrol	56.36	34.13	73.77	56.78	65.78	33.15	43.29

^a^ Compounds are listed in order of elution from an INNOWAX capillary column; Only substances appearing in excess of 1% in at least one blend are shown in the table.

**Table 2:** Pairwise comparison (*t*-test) of IC_50_ values among the top-10 EOs blends with the highest inhibitory activity against the fish pathogen *Aeromonas veronii* bv. *sobria*. Statistically significant differences at *p* < 0.05 are highlighted in bold. The exact composition of each blend is shown in Table S1.

EOs, Blends	#2	#10	#38	#31	#5	#8	#11	#1	#16	#35
**#2**	1.00									
**#10**	**0.03**	1.00								
**#38**	**0.01**	**0.03**	1.00							
**#31**	**0.01**	**0.01**	0.46	1.00						
**#5**	**0.01**	**0.03**	0.51	0.84	1.00					
**#8**	**0.01**	**0.01**	0.23	0.56	0.91	1.00				
**#11**	**0.00**	**0.00**	**0.02**	**0.03**	0.11	**0.03**	1.00			
**#1**	**0.00**	**0.00**	**0.00**	**0.00**	**0.02**	**0.00**	0.09	1.00		
**#16**	**0.00**	**0.01**	**0.02**	**0.03**	0.06	**0.04**	0.28	0.91	1.00	
**#35**	**0.00**	**0.01**	**0.02**	**0.02**	**0.04**	**0.03**	0.13	0.36	0.49	1.00

**Table 3:** Pairwise comparison (*t*-test) with established statistical differences (bold) among the top-10 blends of essential oils (EOs) presenting the highest bactericidal activity against the fish pathogen *Aeromonas veronii* bv. *sobria*. For the composition of the tested EOs, see Table S1 (Supplementary Materials).

EOs, Blends	#2	#10	#38	#31	#5	#8	#11	#1	#16	#35
**#2**	1.000									
**#10**	0.108	1.000								
**#38**	**0.044**	0.151	1.000							
**#31**	**0.006**	**<0.001**	**<0.001**	1.000						
**#5**	**0.022**	**<0.001**	**<0.001**	0.051	1.000					
**#8**	**0.005**	**<0.001**	**<0.001**	0.863	**0.046**	1.000				
**#11**	0.359	0.137	**0.022**	**<0.001**	**<0.001**	**<0.001**	1.000			
**#1**	0.135	0.548	**0.043**	**<0.001**	**<0.001**	**<0.001**	0.208	1.000		
**#16**	**0.013**	**0.017**	0.103	**<0.001**	**<0.001**	**<0.001**	**0.005**	**0.008**	1.000	
**#35**	**0.008**	**0.005**	**0.035**	**<0.001**	**<0.001**	**<0.001**	**0.002**	**0.002**	0.702	1.000

**Table 4:** Greek native medicinal-aromatic plant species used for the extraction of the examined essential oils and origin of the original plant material.

Common Name	Scientific Name (Family)	Cultivation Area
Pennyroyal	^1^ *Mentha pulegium*	Ikaria, SE GR
Greek oregano	^1^*Origanum vulgare* subsp. *hirtum*	Ptolemaida, N GR
Rosemary	^1^ *Rosmarinum officinalis*	Ptolemaida, N GR
Spanish oregano	^1^ *Thymbra capitata*	Ptolemaida, N GR
Savoury	^1^ *Satureja thymbra*	Ikaria, SE GR
Lemon balm	^1^ *Melissa officinalis*	Ptolemaida, N GR
Wild carrot	^2^ *Daucus carota*	* Ikaria, SE GR

* Harvested from the wild; ^1^ Lamiaceae; ^2^ Apiaceae; SE: South-Eastern; N: Northern; GR: Greece.

## Data Availability

Data available on request.

## Supplementary Materials

The following are available online, Table S1: Main constituents and percentage content of essential oils (EOs) from seven Greek native medicinal-aromatic plants (MAPs) tested in vitro against *Aeromonas veronii* bv. *sobria.* *GO: Greek oregano; *S: Savoury; R: Rosemary; WC: Wild carrot; *SO: Spanish oregano; P: Pennyroyal; L: Lemon balm (for the origin of the material see Table 4). The EOs content in bold letters from MAPs marked with asterisk (*) were evaluated as highly effective single-note EOs against the studied fish pathogen (from Anastasiou et al. 2020, with modifications); Figure S1: GC-MS Chromatograms of the most effective bi- and tripartite blends of essential oils (EOs) tested in vitro against *Aeromonas veronii* bv. *sobria* after evaluation of 127 preparations using EOs of seven Greek native medicinal-aromatic plants and their combinations. For the percentage content of the most effective blended essential oil preparations see Table 1. For the identity and origin of each plant, see Table 4; Table S2: Percentage of essential oils (EOs) of seven Greek native medicinal-aromatic plants (MAPs) in the composites prepared (CP) for growth-inhibitory and bactericidal activity testing against the fish pathogen *Aeromonas veronii* bv. *sobria*. GO: Greek oregano, S: Savoury, R: Rosemary, WC: Wild carrot, SO: Spanish oregano, P: Pennyroyal, L: Lemon balm.

## Sample Availability

Samples of the compounds are available on request from the authors.
