# Supplementary material for: Antibacterial Effects of Essential Oils of Seven Medicinal-Aromatic Plants Against the Fish Pathogen Aeromonas veronii bv. sobria: To Blend or Not to Blend?
Source: Molecules. 2021 May 6;26(9):2731. doi: 10.3390/molecules26092731 (PMC8125735; doi:10.3390/molecules26092731)

Supplementary material Figure S1. GC-MS Chromatograms of the most effective bi- and tripartite blends of essential oils (EOs) tested *in vitro* against *Aeromonas veronii* bv. *sobria* after evaluation of 127 preparations using EOs of seven Greek native medicinal-aromatic plants and their combinations. For the percentage content of the most effective blended essential oil preparations see Table 1. For the identity and origin of each plant, see Table 4.

Blend #8: EOs from Greek oregano and savoury (1:1)

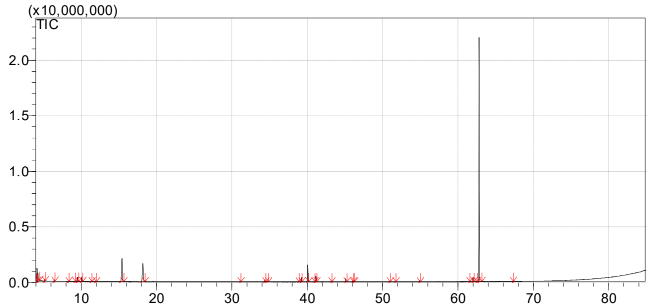

Blend #10: Greek oregano and wild carrot (1:1)

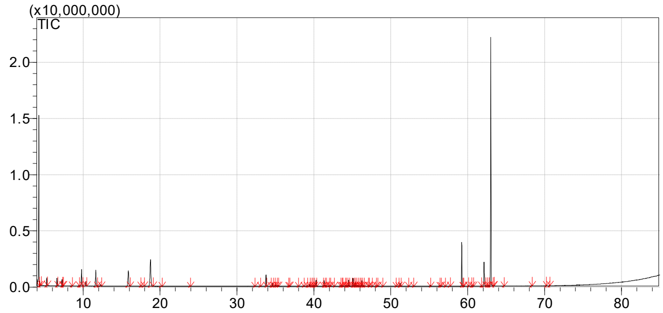

Blend #11: Greek oregano and Spanish oregano (1:1)

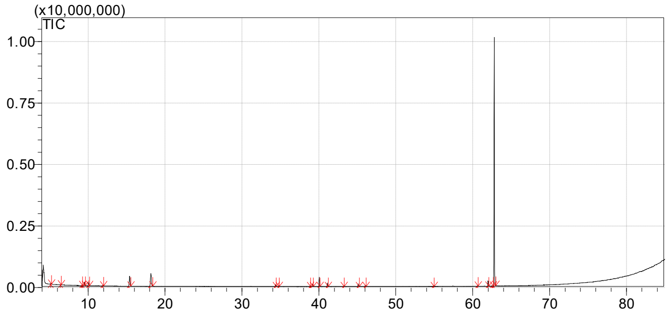

Blend #16: Savoury and Spanish oregano (1:1)

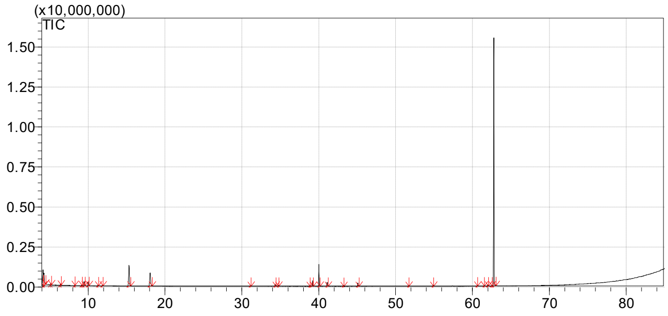

Blend #31: Greek oregano, savoury and Spanish oregano (1:1:1)

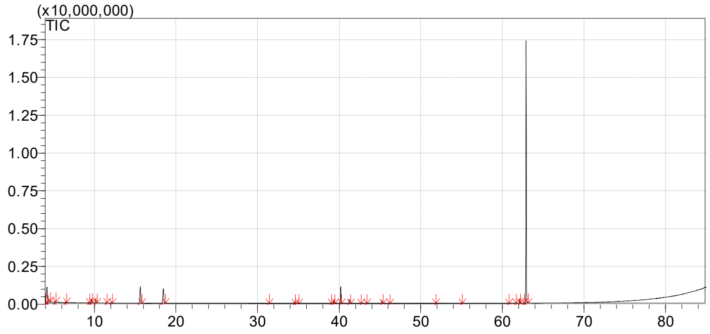

Blend #35: Greek oregano, rosemary and Spanish oregano (1:1:1)

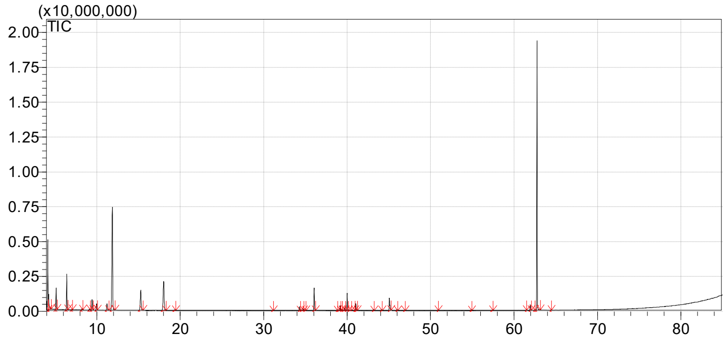

Blend #38: Greek oregano, wild carrot and Spanish oregano (1:1:1)

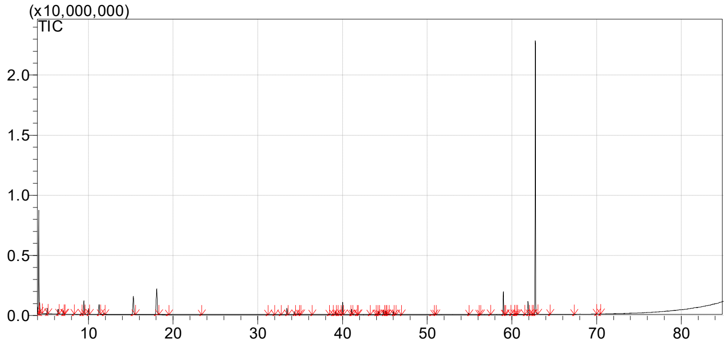

Supplement: Supplementary file 1 [file molecules-26-02731-s001.zip › molecules-1198991-supplementary/SM Figure S1.pdf]
