# Supplementary material for: Antibacterial Effects of Essential Oils of Seven Medicinal-Aromatic Plants Against the Fish Pathogen Aeromonas veronii bv. sobria: To Blend or Not to Blend?
Source: Molecules. 2021 May 6;26(9):2731. doi: 10.3390/molecules26092731 (PMC8125735; doi:10.3390/molecules26092731)
Supplement: Supplementary file 1 [file molecules-26-02731-s001.zip › molecules-1198991-supplementary/SM Table S1.pdf]

Supplementary material Table S1. Main constituents and percentage content of essential oils (EOs) from seven Greek native medicinal-aromatic plants (MAPs) tested *in vitro* against *Aeromonas veronii* bv. *sobria.* ***GO: Greek oregano; *S: Savoury;** R: Rosemary; WC: Wild carrot; ***SO: Spanish oregano;** P: Pennyroyal; L: Lemon balm (for the origin of the material see Table 4). The EOs content in bold from MAPs marked with asterisk (*) were evaluated as highly effective single-note EOs against the studied fish pathogen (from Anastasiou et al. 2020 with modifications).

**No**	**Constituents**	***GO**	***S**	R	WC	***SO**	P	L
**1**	α-Thujene		**2.5**			**3.5**		
**2**	α-Pinene		**1.8**	8.8	20.5	**1.6**		
**3**	Camphene			4.0	1.1			
**4**	Sabinene				2.3			
**5**	β-Pinene		**1.0**	7.6				
**6**	1-Octen-3-ol					**1.0**		
**7**	Myrcene	**1.2**	**2.9**	1.1	6.9	**4.6**		
**8**	3-Octanol						2.0	
**9**	α-Terpinene		**3.5**			**3.9**		
**10**	p-Cymene	**9.6**	**6.5**	1.4		**11.9**		
**11**	Sylvestrene					**1.0**		
**12**	Limonene			1.9	7.5		1.2	
**13**	Eucalyptol			45.0				
**14**	*trans*-Ocimene							1.6
**15**	γ-Terpinene	**5.3**	**34.0**			**20.5**		
**16**	Linalool		**1.4**			**1.1**		
**17**	Camphor			11.5				
**18**	Citronellal							10.2
**19**	Menthone						3.5	
**20**	Isomenthone						5.2	
**21**	Borneol			4.3				
**22**	Terpinen-4-ol					**1.0**		
**23**	α-Terpineol			1.9				
**24**	Citronellol							1.8
**25**	Pulegone						47.6	
**26**	Neral							6.5
**27**	Carvacrolmethylether		**1.8**					
**28**	Piperitone						2.0	
**29**	Methylcitronellate							2.3
**30**	Geranial							8.8
**31**	Bornylacetate			1.9				
**32**	Thymol	**2.1**						
**33**	Carvacrol	**72.0**	**32.8**			**42.0**		
**34**	α-longipinene				5.2			
**35**	Piperitenone						33.0	
**36**	α-Copaene							2.4
**37**	β-Bourbonene							1.5

No	Constituents	*GO	*S	R	WC	*SO	P	L
**38**	β-Caryophyllene		**6.9**	3.7		**3.4**		27.7
**39**	*α*-Caryophyllene							2.2
**40**	*trans*-β-Farnesene							1.2
**41**	γ-Muurolene							12.6
**42**	*α*-Cedrene				3.7			
**43**	Isoeugenol methyl ether				14.8			
**44**	*α*-(*E,E*)-Farnesene							1.8
**45**	γ-Cadinene							1.6
**46**	δ-Cadinene							2.9
**47**	Caryophyllene oxide							2.7
**48**	β-Himachalene				21.6			
**49**	*α*-Cadinol							1.0
