# Supplementary material for: Antibacterial Effects of Essential Oils of Seven Medicinal-Aromatic Plants Against the Fish Pathogen Aeromonas veronii bv. sobria: To Blend or Not to Blend?
Source: Molecules. 2021 May 6;26(9):2731. doi: 10.3390/molecules26092731 (PMC8125735; doi:10.3390/molecules26092731)
Supplement: Supplementary file 1 [file molecules-26-02731-s001.zip › molecules-1198991-supplementary/SM Table S2.pdf]

Supplementary material Table S2. Percentage of essential oils (EOs) of seven Greek native medicinal-aromatic plants (MAPs) in various preparations used for growth-inhibitory and bactericidal activity testing against the fish pathogen *Aeromonas veronii* bv. *sobria*. GO: Greek oregano, S: Savoury, R: Rosemary, WC: Wild carrot, SO: Spanish oregano, P: Pennyroyal, L: Lemon balm.

EOs of MAPs used	Preparation	GO	S	R	WC	SO	P	L
1	1	100.00	-	-	-	-	-	-
	2	-	100.00	-	-	-	-	-
	3	-	-	100.00	-	-	-	-
	4	-	-	-	100.00	-	-	-
	5	-	-	-	-	100.00	-	-
	6	-	-	-	-	-	100.00	-
	7	-	-	-	-	-	-	100.00
2	8	50.00	50.00	-	-	-	-	-
	9	50.00	-	50.00	-	-	-	-
	10	50.00	-	-	50.00	-	-	-
	11	50.00	-	-	-	50.00	-	-
	12	50.00	-	-	-	-	50.00	-
	13	50.00	-	-	-	-	-	50.00
	14	-	50.00	50.00	-	-	-	-
	15	-	50.00	-	50.00	-	-	-
	16	-	50.00	-	-	50.00	-	-
	17	-	50.00	-	-	-	50.00	-
	18	-	50.00	-	-	-	-	50.00
	19	-	-	50.00	50.00	-	-	-
	20	-	-	50.00	-	50.00	-	-
	21	-	-	50.00	-	-	50.00	-
	22	-	-	50.00	-	-	-	50.00
	23	-	-	-	50.00	50.00	-	-
	24	-	-	-	50.00	-	50.00	-
	25	-	-	-	50.00	-	-	50.00
	26	-	-	-	-	50.00	50.00	-
	27	-	-	-	-	50.00	-	50.00
	28	-	-	-	-	-	50.00	50.00
3	29	33.33	33.33	33.33	-	-	-	-
	30	33.33	33.33	-	33.33	-	-	-
	31	33.33	33.33	-	-	33.33	-	-
	32	33.33	33.33	-	-	-	33.33	-
	33	33.33	33.33	-	-	-	-	33.33
	34	33.33	-	33.33	33.33	-	-	-
	35	33.33	-	33.33	-	33.33	-	-
	36	33.33	-	33.33	-	-	33.33	-

EOs of MAPs used	Preparation	GO	S	R	WC	SO	P	L
	37	33.33	-	33.33	-	-	-	33.33
	38	33.33	-	-	33.33	33.33	-	-
	39	33.33	-	-	33.33	-	33.33	-
	40	33.33	-	-	33.33	-	-	33.33
	41	33.33	-	-	-	33.33	33.33	-
	42	33.33	-	-	-	33.33	-	33.33
	43	33.33	-	-	-	-	33.33	33.33
	44	-	33.33	33.33	33.33	-	-	-
	45	-	33.33	33.33	-	33.33	-	-
	46	-	33.33	33.33	-	-	33.33	-
	47	-	33.33	33.33	-	-	-	33.33
	48	-	33.33	-	33.33	33.33	-	-
	49	-	33.33	-	33.33	-	33.33	-
	50	-	33.33	-	33.33	-	-	33.33
	51	-	33.33	-	-	33.33	33.33	-
	52	-	33.33	-	-	33.33	-	33.33
	53	-	33.33	-	-	-	33.33	33.33
	54	-	-	33.33	33.33	33.33	-	-
	55	-	-	33.33	33.33	-	33.33	-
	56	-	-	33.33	33.33	-	-	33.33
	57	-	-	33.33	-	33.33	33.33	-
	58	-	-	33.33	-	33.33	-	33.33
	59	-	-	33.33	-	-	33.33	33.33
	60	-	-	-	33.33	33.33	33.33	-
	61	-	-	-	33.33	33.33	-	33.33
	62	-	-	-	33.33	-	33.33	33.33
	63	-	-	-	-	33.33	33.33	33.33
	64	25.00	25.00	25.00	25.00	-	-	-
	65	25.00	25.00	25.00	-	25.00	-	-
	66	25.00	25.00	25.00	-	-	25.00	-
	67	25.00	25.00	25.00	-	-	-	25.00
	68	25.00	25.00	-	25.00	25.00	-	-
	69	25.00	25.00	-	25.00	-	25.00	-
	70	25.00	25.00	-	25.00	-	-	25.00
	71	25.00	25.00	-	-	25.00	25.00	-
**4**	72	25.00	25.00	-	-	25.00	-	25.00
	73	25.00	25.00	-	-	-	25.00	25.00
	74	25.00	-	25.00	25.00	25.00	-	-
	75	25.00	-	25.00	25.00	-	25.00	-
	76	25.00	-	25.00	25.00	-	-	25.00
	77	25.00	-	25.00	-	25.00	25.00	-
	78	25.00	-	25.00	-	25.00	-	25.00
	79	25.00	-	25.00	-	-	25.00	25.00

EOs of MAPs used	Preparation	GO	S	R	WC	SO	P	L
	80	25.00	-	-	25.00	25.00	25.00	-
	81	25.00	-	-	25.00	25.00	-	25.00
	82	25.00	-	-	25.00	-	25.00	25.00
	83	25.00	-	-	-	25.00	25.00	25.00
	84	-	25.00	25.00	25.00	25.00	-	-
	85	-	25.00	25.00	25.00	-	25.00	-
	86	-	25.00	25.00	25.00	-	-	25.00
	87	-	25.00	25.00	-	25.00	25.00	-
	88	-	25.00	25.00	-	25.00	-	25.00
	89	-	25.00	25.00	-	-	25.00	25.00
	90	-	25.00	-	25.00	25.00	25.00	-
	91	-	25.00	-	25.00	25.00	-	25.00
	92	-	25.00	-	25.00	-	25.00	25.00
	93	-	25.00	-	-	25.00	25.00	25.00
	94	-	-	25.00	25.00	25.00	25.00	-
	95	-	-	25.00	25.00	25.00	-	25.00
	96	-	-	25.00	25.00	-	25.00	25.00
	97	-	-	25.00	-	25.00	25.00	25.00
	98	-	-	-	25.00	25.00	25.00	25.00
	99	20.00	20.00	20.00	20.00	20.00	-	-
	100	20.00	20.00	20.00	20.00	-	20.00	-
	101	20.00	20.00	20.00	20.00	-	-	20.00
	102	20.00	20.00	20.00	-	20.00	20.00	-
	103	20.00	20.00	20.00	-	20.00	-	20.00
	104	20.00	20.00	20.00	-	-	20.00	20.00
	105	20.00	20.00	-	20.00	20.00	20.00	-
	106	20.00	20.00	-	20.00	20.00	-	20.00
	107	20.00	20.00	-	20.00	-	20.00	20.00
	108	20.00	20.00	-	-	20.00	20.00	20.00
**5**	109	20.00	-	20.00	20.00	20.00	20.00	-
	110	20.00	-	20.00	20.00	20.00	-	20.00
	111	20.00	-	20.00	20.00	-	20.00	20.00
	112	20.00	-	20.00	-	20.00	20.00	20.00
	113	20.00	-	-	20.00	20.00	20.00	20.00
	114	-	20.00	20.00	20.00	20.00	20.00	-
	115	-	20.00	20.00	20.00	20.00	-	20.00
	116	-	20.00	20.00	20.00	-	20.00	20.00
	117	-	20.00	20.00	-	20.00	20.00	20.00
	118	-	20.00	-	20.00	20.00	20.00	20.00
	119	-	-	20.00	20.00	20.00	20.00	20.00
	120	16.67	16.67	16.67	16.67	16.67	16.67	-
**6**	121	16.67	16.67	16.67	16.67	16.67	-	16.67
	122	16.67	16.67	16.67	16.67	-	16.67	16.67

EOs of MAPs used	Preparation	GO	S	R	WC	SO	P	L
	123	16.67	16.67	16.67	-	16.67	16.67	16.67
	124	16.67	16.67	-	16.67	16.67	16.67	16.67
	125	16.67	-	16.67	16.67	16.67	16.67	16.67
	126	-	16.67	16.67	16.67	16.67	16.67	16.67
**7**	127	14.29	14.29	14.29	14.29	14.29	14.29	14.29
